# A Study on Surface Hardening and Wear Resistance of AISI 52100 Steel by Ultrasonic Nanocrystal Surface Modification and Electrolytic Plasma Surface Modification Technologies

**DOI:** 10.3390/ma16206824

**Published:** 2023-10-23

**Authors:** Nurtoleu Magazov, Zarina Satbaeva, Bauyrzhan Rakhadilov, Auezhan Amanov

**Affiliations:** 1 Department of Mechanical Engineering, Daulet Serikbayev East Kazakhstan Technical University, Ust-Kamenogorsk 070010, Kazakhstan; magazovn@gmail.com; 2 Surface Engineering and Tribology Research Center, Sarsen Amanzholov East Kazakhstan University, Ust-Kamenogorsk 070002, Kazakhstan; 3 PlasmaScience LLP, Ust-Kamenogorsk 070010, Kazakhstanrakhadilovb@gmail.com (B.R.); 4 Department of Mechanical Engineering, Sun Moon University, Asan 31460, Republic of Korea; 5 Faculty of Engineering and Natural Sciences, Tampere University, 33720 Tampere, Finland

**Keywords:** AISI 52100 steel, wear resistance, ultrasonic nanocrystal surface modification, electrolytic-plasma surface modification

## Abstract

In this study, a surface hardening of AISI 52100 bearing steel was performed by ultrasonic nanocrystal surface modification (UNSM), and electrolytic-plasma thermo-cyclic surface modification (EPSM), and their effects on the wear resistance were investigated. To evaluate the impact of these treatments on the wear resistance, the friction tests under dry conditions were conducted using a ball-on-disk tribometer in accordance with ASTM G99. The microstructure of the samples before and after treatment was characterized by scanning electron microscopy. The micro-hardness with respect to the depth from the top surface was measured using a Vickers micro-hardness tester. Microstructural observations showed that EPSM treatment led to the formation of residual austenite in the surface layer, while UNSM treatment led to the formation of a surface severe plastic deformation layer on the surface of the samples. The increase in the micro-hardness of the treated layer was confirmed after UNSM at room temperature and after EPSM at different cycles. The highest increase in wear resistance was observed for the specimen treated by UNSM treatment at 700 °C and five cycles of EPSM treatment. In addition, the wear volume, which has correlation with the friction coefficient and hardness, was determined.

## 1. Introduction

Bearing steel is one of the key materials in designing and manufacturing various machine parts. Therefore, this type of steel must satisfy high requirements, including wear resistance, strength, and contact hardness [1,2]. To meet these expectations, researchers are constantly exploring new designs or improving existing ones, optimizing the microstructure and characteristics of bearing materials. To improve the performance of bearing materials, the application of modern technologies for hardening the surface layer of metallic materials has been increasingly studied recently. Thus, the surface layer largely determines the properties of bearings.

Currently, surface modification methods using concentrated energy flows such as laser, electron, ion beams, and plasma flows are widely used [3]. Treatment methods are based on the use of concentrated energy flows use high heating and cooling rates and very short material holding times at high temperatures. During the treatment with concentrated energy flows, thermal and impact mechanical effects can be simultaneously realized due to the formation of a high-temperature gradient on the surface and in the near-surface layers of the treated material.

One of the promising methods of steel surface treatment with concentrated energy flows is an electrolytic-plasma thermo-cyclic surface modification (EPSM), forming a fine-grained martensitic structure which is beneficial for both the mechanical and tribological properties [4,5]. EPSM has another characteristic of creating residual stresses in the material by treating its surface in certain areas. When localized heating of the material is carried out, bonds between the heated and cooled areas occur, which prevent free expansion of the material. This results in stresses that can cause both elastic and plastic deformations of the material. Phase and structural transformations occurring with an increase in specific volume during cooling lead to the appearance of residual compressive stresses on the surface, which increase the strength characteristics of steel [6]. Thus, it can be stated that one of the reasons for the hardening of steels by EPSM technology is an impact-mechanical effect, which usually occurs during surface treatment by plastic deformation, electrospark alloying, ultrasonic treatment, etc. However, no comparative studies have been conducted on EPSM with others, particularly ultrasonic treatment.

The ultrasonic nanocrystal surface modification (UNSM) treatment is a mechanical impact method which forms a nanostructured surface layer with high mechanical properties and a high damping ability on the surface of materials. In turn, this layer protects the surface from being worn out and damaged [7]. Moreover, the UNSM technology refines the coarse grains into nano-grains, increases the dislocation density, and segregates surface carbides [8,9].

In this study, AISI 52100 steel specimens treated by UNSM and EPSM technologies and their effects on wear resistance under dry sliding conditions against a 100Cr6 alloy counterpart were investigated. The main objective of this study is to understand the role of each UNSM and EPSM technology on the microstructural changes, phase composition, micro-hardness, and wear resistance of AISI 52100 bearing steel.

## 2. Materials and Methods

The samples made of AISI 52100 steel with a diameter of 25 mm and a thickness of 5 mm were used in this study. This bearing steel is mainly used for manufacturing aircraft bearings and other highly loaded parts. Usually, this steel is preferably vacuum arc remelted for optimum performance. The chemical composition and mechanical properties of the samples are listed in Table 1.

**Figure 1:**
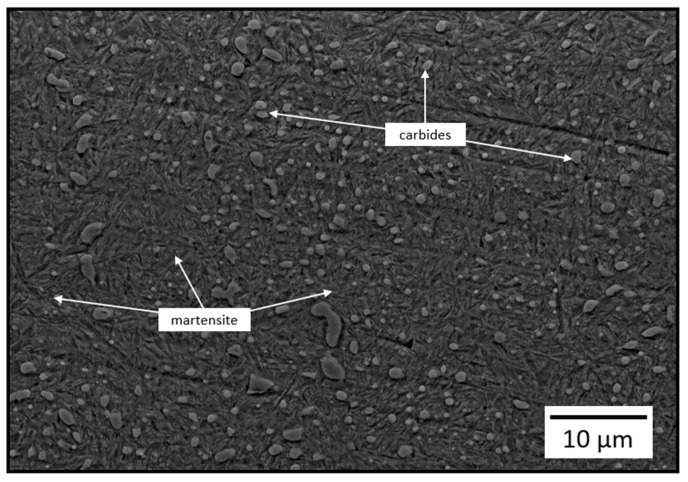
Cross-sectional microstructure of AISI 52100 bearing steel.

Figure 1 shows the typical microstructure of AISI 52100 steel. It consists of martensite and carbides. As shown in Figure 1, the carbide particles are uniformly distributed in the matrix and close to a regular spherical shape.

**Figure 2:**
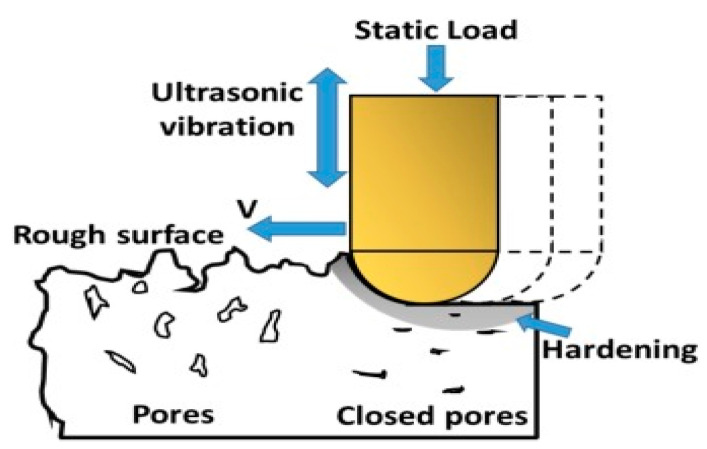
Schematic view of UNSM treatment.

UNSM is a mechanical surface hardening technique that has been applied to metallic materials [7]. In the UNSM (see Figure 2), a tip made of WC with a diameter of 2.38 mm is attached to an ultrasonic device to generate high-frequency strikes at 20,000 times per second. The amplitude and frequency of the striking force are controlled via an ultrasonic transducer. The striking impact consists of static and dynamic loads, which can be independently adjusted depending on the treatment target such as plastic deformation, surface smoothing, and grain size. The UNSM treatment parameters at RT and HT are listed in Table 2. The samples were heated up using a halogen lamp with a power of 1 kW. During UNSM treatment, the distance between the sample and the halogen lamp was about 15 cm. The samples were heated up to 500 °C. The actual temperature of the samples was measured using a pyrometer (IMPAC, IGA140, LumaSense Technologies, Ballerup, Denmark). More details of UNSM at the HT setting can be found in a previous study [10].

**Figure 3:**
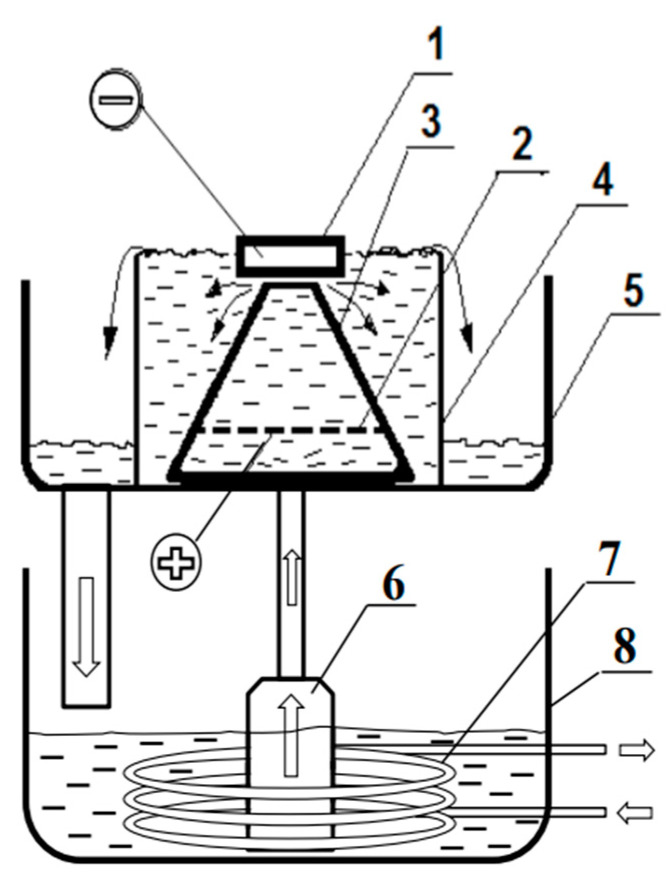
Schematic diagram of the EPSM unit. 1—sample to be treated (cathode), 2—stainless steel anode with holes, 3—cone-shaped partition, 4—electrolytic cell, 5—pan, 6—pump, 7—heat exchanger, and 8—bath with electrolyte.

EPSM is a type of steel surface hardening technology. The EPSM process involves high-speed heating of the surface layer under the influence of plasma formed between the electrolyte and the sample, followed by cooling in the electrolyte. Based on the electrolyte-plasma method, it is possible to carry out oxidizing, polishing, chemical–thermal treatment, and hardening of the surface layer of the material [11,12]. EPSM radically affects the structural and phase transformations in steels, which improves the quality of the part and gives them the desired properties. EPSM of AISI 52100 steel was carried out in the cathodic mode at the electrolytic-plasma treatment unit as schematically shown in Figure 3.

The power source was a rectifier giving a maximum output value of 360 V/100 A in a direct current. An aqueous solution containing 10% sodium carbonate (Na_2_CO_3_) was used as an electrolyte. In thermo-cyclic EPSM, the sample experiences heating and cooling processes depending on the process modes. The voltage magnitude (U) and treatment time (t) were varied in one cycle and repeated at different cycles for each sample. The EPSM regimes are listed in Table 3. When a voltage of 320 V is applied, the sample is heated rapidly, and when the voltage is reduced to 50 V, the sample is cooled relatively slowly. In this process, cooling can occur at different rates due to the temperature of the electrolyte, which is varied by the applied voltage.

The phase composition was determined on an X’Pert Pro X-ray diffractometer (PANalytical, Amsterdam, Netherlands) with CuKa radiation at a voltage of 40 kV, a current of 30 mA, scanning parameters at 35° < 2θ < 85°, a step of 0.02°, and an exposure time of 5 s. The microstructure of the samples was revealed by chemical etching using a 4% solution of nitric acid (HNO_3_) in ethyl alcohol. This reagent was proposed long ago and remains one of the most widely used in metallographic practice. Microstructure was characterized using a scanning electron microscope Mira 3 (TESCAN, Brno, Czech Republic). The samples were fixed on conductive tape to prepare the study material. Since the investigated samples were on epoxy, the samples were coated with a conductive carbon layer on a Quorum Q150R ES sputtering machine. The study was carried out at an accelerating voltage of 20 kV. The average grain size/diameter was determined by measuring grains in micrographs of the sample cross-section at 10k magnification, taken from different areas of the sample. The images were processed for grain size measurement using the ImageJ 1.54d program. The micro-hardness distribution by depth was also measured using a Vicker’s micro-hardness tester (Metolab 502, St. Petersburg, Russia) equipped with a diamond indenter and a load cell up to 1000 g. For the measurement, a load of 200 g and a dwell time of 10 s were used [13]. Tribological studies were conducted on a TRB^3^ tribometer (Anton Paar GmbH, Graz, Austria) using a ball–disk configuration in accordance with ASTM G99 [14]. The amount of wear on the samples was investigated by contact profilometry from the wear trace profiles using a Profilometer 130 (Proton, Zelenograd, Russia). For each wear trace, six cross-sectional profiles were obtained at equal intervals and used to calculate the wear volume loss values.

## 3. Results and Discussion

**Figure 4:**
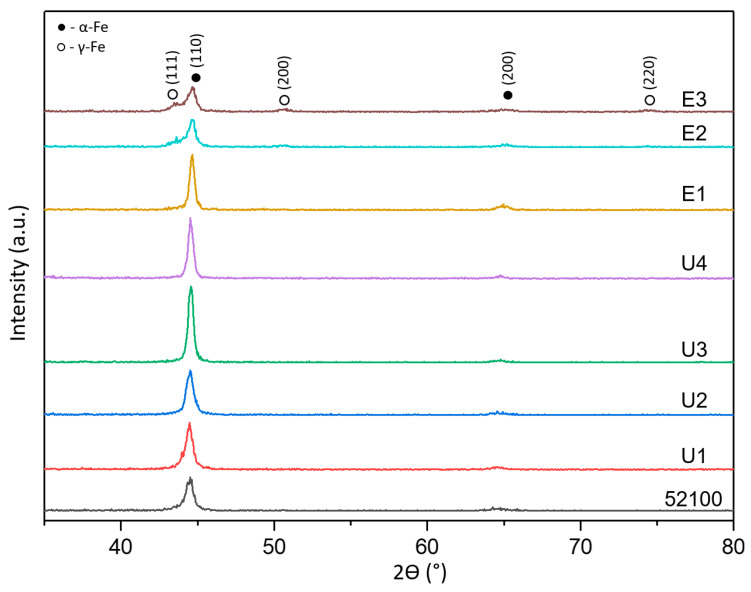
XRD patterns of AISI 52100 steel treated by UNSM and EPSM methods.

Figure 4 depicts the X-ray diffraction patterns of AISI 52100 steel treated by UNSM and EPSM methods. As observed, the X-ray peaks are predominantly attributed to the α-Fe phase. Following surface modification, the X-ray peaks slightly shift towards higher angles. This indicates an increased lattice deformation due to thermo-mechanical effects after the UNSM and EPSM treatments. For certain peaks, a broadening of the diffraction peak (full width at half maximum—FWHM) was observed due to the high lattice micro-strain and refined grain size. The resulting plastic deformation on the surface of the steel leads to an increase in the density of defects, such as dislocations, causing peak broadening [15,16]. Furthermore, a decrease in the intensity of the (110) peak after 3 and 5 cycles of EPSM was noted. This indicates a phase transformation and the appearance of γ-Fe phases (111), (200), and (220), which may suggest the presence of residual austenite in the steel structure.

**Figure 5:**
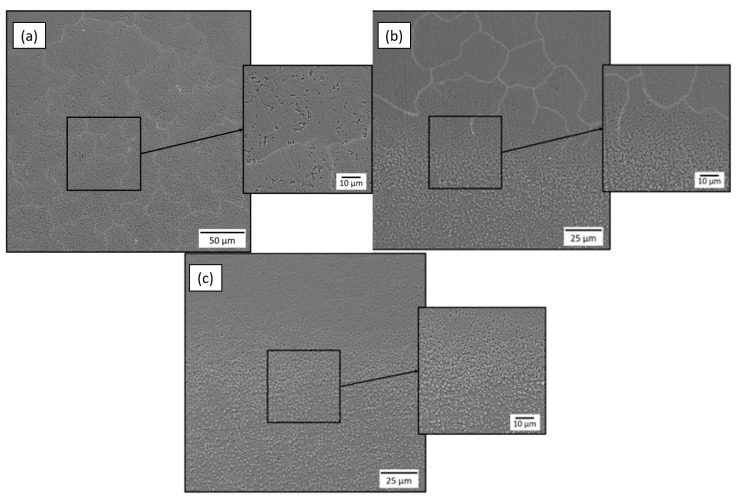
Microstructure of the modified layer of AISI 52100 steel samples treated by EPSM at one (**a**), three (**b**), and five (**c**) cycles.

**Figure 6:**
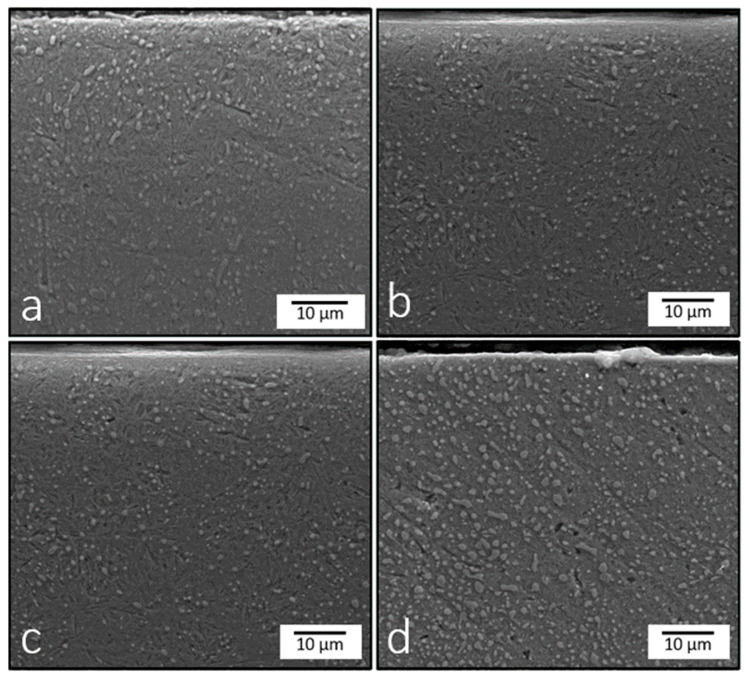
Microstructure of AISI 52100 steel samples treated by UNSM at different temperatures: RT (**a**), 300 °C (**b**), 500 °C (**c**), and 700 °C (**d**).

Changes in the microstructure of the material due to heat and mechanical treatment are the main reasons for improving the mechanical properties of metallic materials. Therefore, a microstructural study is carried out to identify the changes in the structure after the treatments. Cross-sections of the samples before and after the treatments were taken for analysis. The samples were prepared using conventional sectioning methods followed by grinding, mechanical polishing, and etching. The samples were ground and polished using an abrasive SiC paper with grit sizes ranging from 100C to 2000C, as well as velvet cloth. They were etched with Nital containing 4% HNO_3_ for 7 s, cleaned with alcohol, and air dried. The SEM images after the EPSM and UNSM treatments are shown in Figure 5 and Figure 6, respectively. 

In Figure 5, the typical structure of AISI 52100 steel after EPSM treatment can be observed with the presence of three zones: treated layer, transition layer, and initial layer. It can be seen that as the number of EPSM cycles increases, the boundary of the diffusion layer becomes more clearly defined. According to the obtained images, it is possible to observe the appearance of residual austenite in the microstructure of AISI 52100 steel, which is also confirmed by the appearance of the corresponding peaks in XRD (see Figure 4). During EPSM, the key parameter is the periodic temperature increase at which the metal reaches a value above the α→γ phase transition point, known as the austenitization process [17]. This causes ferrite and cementite to dissolve, forming austenite, which creates space for carbon atoms. Cooling after the process causes the iron atoms to change their arrangement to each other, converting the γ-phase back to the α-phase due to the high cooling rate by the electrolyte. The rapid cooling interferes with the proper distribution of carbon to form pearlite or bainite, resulting in the formation of martensite and residual austenite. Combining these two phases gives the steel unique mechanical characteristics, such as the high hardness of martensite and the higher ductility provided by austenite.

As for the UNSM treatment, no phase transformations occurred, but changes after modification can be observed. For the UNSM samples at 300 °C (Figure 6b), 500 °C (Figure 6c) and 700 °C (Figure 6d), a formed dense S^2^PD layer with a thickness not exceeding 5 μm can be observed on the top surface. This is typical for UNSM treatment, which can lead to grain size reduction and severe plastically deformed layers [18]. Additionally, a reduction in carbide size is observed: the average size decreased from 627 nm in the original sample to 217 nm, 280.5 nm, 350.7 nm, and 337 nm on the top surface of the samples after UNSM at RT, 300 °C, 500 °C, and 700 °C, respectively. The fine carbides can contribute not only to the uniform load distribution but also reduce the possibility of the formation of local stress concentrations, which further increases the wear resistance of the material under mechanical stress conditions.

**Figure 7:**
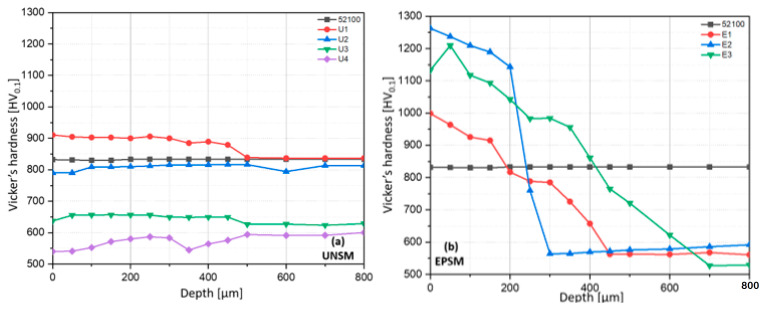
Comparison of Vicker’s hardness of AISI 52100 steel for UNSM (**a**) and EPSM (**b**) depending on depth from the top surface.

The effects of UNSM and EPSM methods on the micro-hardness of AISI 52100 steel are presented in Figure 7. Among the UNSM-treated samples (Figure 7a), the sample treated at RT increased the hardness of AISI 52100 by about 10% in the near-surface region. The hardness profile obtained from the UNSM treatment affected the hardness up to a depth of 400 µm below the surface. However, it is noted that a decrease in micro-hardness was observed with an increase in treatment temperature. The micro-hardness of the samples treated by UNSM at 300 °C, 500 °C, and 700 °C decreased across the volume by 5%, 24%, and 36%, respectively. These results are similar to those in a previous paper [19]. For the EPSM-treated samples (Figure 7b), the micro-hardness measurement results showed that the hardness of the samples mainly increased in the heat-treated zone for all three treatment regimes. The increase in micro-hardness was not uniform in depth. The sample subjected to three cycles of EPSM treatment (E2) showed the highest hardness with a maximum value of 1260 HV. The higher hardness of the E2 sample may be due to more intense phase transformations in the martensitic structure due to fewer EPSM cycles. These results in a more complete transformation of austenite to martensite and probably a lower residual austenite content, which together increases the overall hardness of the material. However, the sample treated with five cycles (E3), while having a higher depth of treatment, may exhibit higher ductility and fracture resistance than E2. This may be due to a more uniform distribution of residual austenite and the optimization of phase structures, which contributes to the improved mechanical performance of the material. 

In addition, we can observe a decrease in the overall microhardness at HT-UNSM and microhardness in the matrix at all EPSM regimes. This can be explained by a phenomenon known as the “un-strengthening effect”. In certain heat treatments, such as tempering, hardened steels are partially restored to their initial properties. The rate of un-strengthening is largely determined by the heating temperature of the metal and the degree of hardening. This effect can be observed in the tempering of high-carbon steel at different temperatures, and its increase has a negative effect on the hardness of bearing steels [20]. Additionally, it is crucial to note that this un-strengthening effect coincides with an increase in grain size and structural alterations, contributing to the overall changes in the mechanical properties of the material.

**Figure 8:**
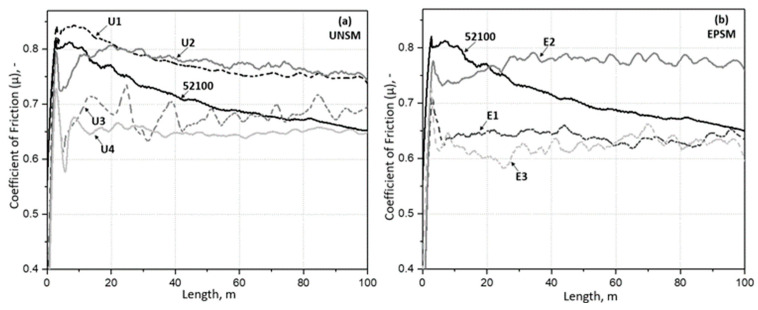
Comparison of COF as a function of sliding distance for AISI 52100 steel samples after UNSM (**a**) and EPSM (**b**) treatments.

For tribological tests, the ball–disk method was used, in which the counterbody was a 100Cr6 ball with a diameter of 6 mm. The tests were conducted in an ambient environment with a sliding distance of 100 m, a relative sliding velocity of 4 cm/s, and a normal load of 7 N. The results of the variation in the coefficient of friction (COF) as a function of sliding distance are shown in Figure 8. The interaction of sliding pairs consisting of bearing steel samples treated with the UNSM (Figure 8a) and EPSM (Figure 8b) treatments showed different values of COF. All samples demonstrated a “running-in zone” in which there was a leveling of high irregularities, adhesion of the sample and counterbody surfaces, and wear of the original surface film. These changes can both increase and decrease the COF. The initial sample and the sample after UNSM at RT (U1) showed similar friction curves. The COF of the initial and U1 samples first reached maximum values and then gradually decreased to 0.65 and 0.75, respectively, throughout the sliding distance (Figure 8a). The samples after UNSM at RT (U2, U3 and U4) also showed a similar friction behavior comparing to the initial and U1 samples, but the COF decreased significantly after reaching the maximum values of COF. Interestingly, it started gradually increasing before relative stabilization occurred. The U1 and U2 samples have a very similar friction behavior after a sliding distance of 20 m, while the stabilization of the U3 sample took longer than that of the U4 sample. Despite the detrimental hardness values of the U3 and U4 samples, they demonstrated a lower COF than the U1 and U2. Hence, it can be concluded that the surface roughness of the samples after UNSM treatment was a dominant factor in controlling the COF. It has been previously observed that UNSM treatment decreases the COF of metallic materials, which can be attributed to the characteristics of the UNSM treatment such as improving the surface integrity, refining the grain size and the presence of micro-dimples on the surface, etc. [8]. In the case of the samples after EPSM treatment, there was a noticeable decrease in the COF for both one (E1) and five (E3) treatment cycles (Figure 8b). The sample treated in three cycles (E2) of EPSM showed the highest COF among all the samples in the initial period. Further, the COF sharply decreased and then gradually increased during the transition period and finally stabilized at 0.77 at a steady state. Similar values and friction curves were observed for the E1 and E3 samples, where a running-in zone was also present, gradually decreasing until the COF stabilized during the steady state period at 0.64 and 0.62, respectively.

**Figure 9:**
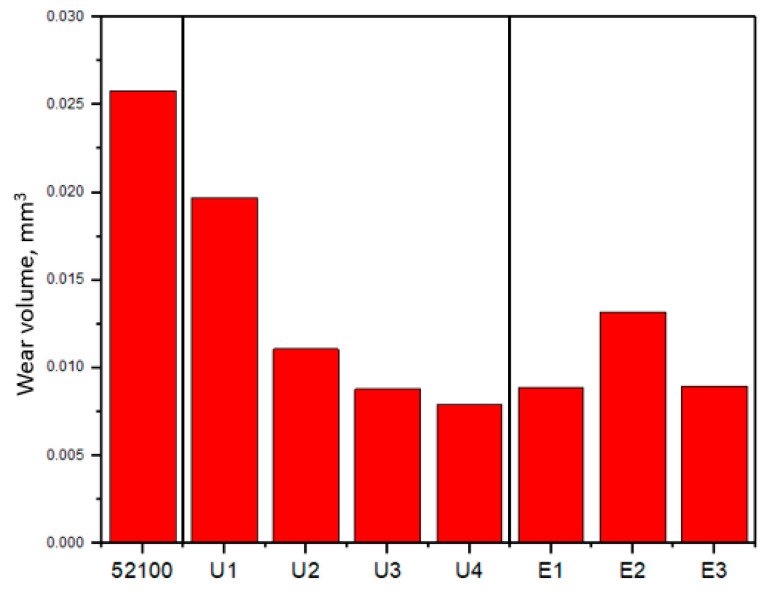
Comparison of wear volume of AISI 52100 steel after UNSM and EPSM treatments.

**Figure 10:**
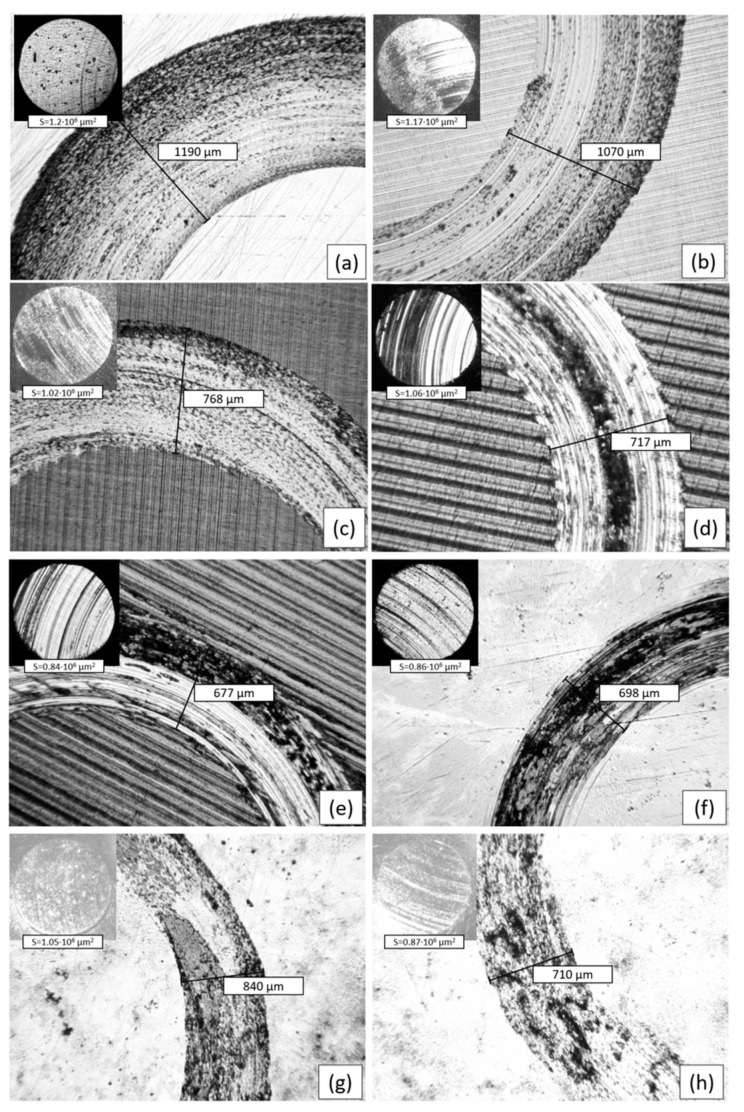
Worn surfaces of samples: (**a**) initial steel, (**b**) U1, (**c**) U2, (**d**) U3, (**e**) U4, (**f**) E1, (**g**) E2, and (**h**) E3 against the counterbody shown in the upper left corner.

Figure 9 and Figure 10 show the wear volume values and optical micrographs of the wear track on the sample and the wear scar on the counterbody after the friction test, respectively. A direct relationship was observed between the COF values, wear volume, and wear track width. It is obvious that the samples with high COF values, especially in the running-in zone, have more increased wear. It can also be observed that lower wear is inherent in samples with the presence of a more steady-state condition [21]. From the results, it was determined that the sample at RT (U1) had the highest wear loss with UNSM treatment and the lowest at 700 °C (U4). At the same time, after EPSM treatment, the highest wear loss was observed for the sample treated for three cycles (E2) and the lowest at five cycles (E3). The width of the worn surface in the initial and U1 samples showed close values (1190 μm and 1070 μm) corresponding to the wear volume values. At the same time, after UNSM at HT, the width of the worn surface decreases significantly and reaches a minimum value of 677 μm for sample U4. Similarly, for the samples after EPSM for all three regimes, the same significant reduction in the wear width was observed, where the lowest value is for sample E1 with a groove width of 698 µm. The value of the worn surface width correlates with the COF and wear volume values for all samples. The worn surface of the samples shows clear abrasion marks and smeared fragment layers. Material transfer and spalling occurred during sliding friction between the two contact surfaces. Adhesive wear leads to the formation of a certain number of abrasive particles, which results in abrasive wear. Thus, the wear mechanisms of the samples were predominantly adhesive wear and abrasive wear of varying degrees. The worn tip of the counter body has a regular round shape.

The correlation of wear values with the hardness of the samples is clearly shown. A higher value of wear volume was obtained for the samples after UNSM and EPSM with high surface hardness (Figure 7). Wang et al. investigated the wear behavior of AISI 52100 steel with different types of structures [22]. They pointed out that under severe wear conditions, the initial structure with a higher hardness does not show better wear resistance. It was also determined that with an increasing load, the amount of wear decreased for the samples with lower hardness.

## 4. Conclusions

An in-depth analysis of the effect of UNSM and EPSM technologies on the microstructural and tribological properties of AISI 52100 steel has been carried out. X-ray diffraction analysis revealed minor changes in the diffraction peaks, indicating increased lattice deformation due to thermomechanical effects after the UNSM and EPSM treatments. The microstructural study showed the formation of different zones after EPSM treatment, with a more clearly defined diffusion layer boundary with the increasing the number of cycles.

It is important to note that austenitization during the EPSM process promotes the formation of martensite and residual austenite during rapid cooling. Both technologies significantly affect the surface and microstructure of AISI 52100 steel. Reduced grain size and improved surface integrities reduced the friction coefficients. However, there are also notable differences. EPSM causes austenitization and phase changes, while UNSM leads to the formation of a dense S^2^PD layer. An important aspect of the study is the potential for combining UNSM and EPSM technologies.

This approach provides a unique opportunity to optimize the microstructure and mechanical properties of the material at different depths and in different layers. For example, it is possible to combine EPSM treatment to induce austenitization and create martensite and residual austenite, followed by UNSM treatment to form a dense surface layer. A combined approach combining UNSM and EPSM exposure represents a promising strategy for creating materials with optimal mechanical and tribological characteristics. These methods can be applied either individually or jointly, depending on specific requirements and production goals. The results of this study have important practical implications for the specialists involved in the development and production of bearing materials.

## Figures and Tables

**Table 1:** Chemical composition in wt.% and mechanical properties of the samples used in this study. (YM—Youngs modulus; YP—yield point; TS—Tensile strength).

Material	Chemical Composition										Mechanical Properties		
	C	Si	Mn	P	S	Cu	Ni	Cr	Mo	Fe	YM, GPa	YP, GPa	TS, GPa
AISI 52100	1.03	0.26	0.34	0.01	0.008	0.09	0.06	1.4	0.03	rest	208	1.37	1.57–1.96

**Table 2:** UNSM treatment parameters of AISI 52100 steel at room and high temperatures (RT and HT).

Sample	Frequency, kHz	Amplitude, µm	Load, N	Interval, µm	Ball Diameter, mm	Treatment Temperature, °C
U1	20	30	20	10	2.38	RT
U2						300
U3						500
U4						700

**Table 3:** EPSM treatment parameters at different cycles.

Sample	1 Cycle				Number of Cycles
	U_1_, V	t_1_, s	U_2_, V	t_2_, s	
E1	380	2	50	7	1
E2	380	2	50	7	3
E3	380	2	50	7	5

## Data Availability

Not applicable.
